# Antecedents of organizational citizenship behavior among Iranian nurses: a multicenter study

**DOI:** 10.1186/s13104-015-1505-1

**Published:** 2015-10-08

**Authors:** Fakhredin Taghinezhad, Mahboobe Safavi, Afsaneh Raiesifar, Sayed Hossein Yahyavi

**Affiliations:** School of Nursing and Midwifery, Islamic Azad University, Tehran Medical Sciences Branch, Tehran, Iran; Department of Nursing, Farabi hospital, Tehran University of Medical Sciences (TUMS), Tehran, Iran; Department of Nursing Management, School of Nursing and Midwifery, Islamic Azad University, Tehran Medical Sciences Branch, Tehran, Iran; School of Nursing and Midwifery, Tehran University of Medical Sciences, Tehran, Iran

**Keywords:** Organizational citizenship behaviors, Organizational commitment, Job satisfaction, Procedural justice, Nursing

## Abstract

**Background:**

Organizational citizenship behavior (OCB) improves efficiency and employees’ participation and generally provides a good ambiance. This study was conducted to determine the role of job satisfaction (JS), organizational commitment (OC) and procedural justice (PJ) in explaining OCB among nurses working in fifteen educational-treatment centers in Tehran-Iran, to provide guidelines for health care managers’ further understanding of how to encourage citizenship behavior among nurses.

**Methods:**

In this multi-center descriptive-correlational study 373 nurses were evaluated through a Multi-stage cluster sampling method after obtaining approval from the Ethics Committee of Islamic Azad University, Tehran Medical Branch and Tehran University of Medical Sciences Research Deputy. Nurses who signed the informed consent and holding a bachelor or master degree, having a minimum one year of job experience and not having organizational management position during the questionnaire distribution were included in the study. In order to collect data, Demographic questionnaire, Podsakoff et al. (Leadersh Q 1(2):107–142, 1990) OCB questionnaire, OC questionnaire, Aelterman et al. (Educ Stud 33(3):285–297, 2007) JS questionnaire and PJ questionnaire were used. These questionnaires were translated into Persian and content validity was confirmed by an expert group; their reliability was calculated by the internal consistency Cronbach alpha coefficient and it was satisfied. Data were analyzed by descriptive statistics, Comparative mean tests, correlation coefficient and multiple-regression in the SPSS software version 11.

**Result:**

The general mean and all five aspects of OCB that ranked higher than 3 were evaluated in a “quite desired” state. The mean for perceived procedural justice, the general mean for JS and the mean of general grade for OC from the nurses’ was in “quite desired” state. Finding from multiple regression indicated that OC and PJ exhibit about 19 % of OCB variance totally which is statistically significant (P < 0.01). JS had no significant impact on explaining OCB.

**Conclusion:**

OC was the strongest predictor of nurses’ OCB followed by perceived procedural justice. So, improving these factors can initiate better citizenship behavior among nurses.

## Background

In the long run, an effective organization is distinguished from a non-effective one by its employees [1]. Since these people find themselves belonging to the organization, they would channel all their efforts and do their best for the success of their institution and fulfillment of the organizational objectives. These unprecedented, voluntary, helpful and effective efforts are interpreted differently, the most prevalent being Organizational citizenship behaviors (OCB) [2]. The OCB improves efficiency and employees’ participation, it encourages teamwork and inter-organization cooperation, also, it reduces the costs of mistakes and generally provides a good work environment [3]. Among the organizations in which the issue of OCB seems critical are health and treatment services centers. Treatment and hospital systems are continuously experiencing new changes which influence the way through which patients and clients receive services, however, many hospital centers are not capable of confronting these unwanted changes [4, 5]. It is believed that, had the employees of these centers possessed a higher level of OCB, there would have been a better chance for these centers to remain competitive. This is because OCB sustains reinforcement in creativity and improvement through meeting the new needs of the organization and the clients [4, 5]. From one side, seniors population growth, technological advances, welfare issues and increase in expectations of patients in the quality of caring in the field of nursing added to a growing shortage in the nursing work force in general, and intention to leave service in the existing work force in particular has increased the importance of OCB among nurses [6, 7]. These facts have turned the optimized use of existing human capital into a major concern for managers in the field [6, 7]. Also, the clinic nurses, as the largest professional group working in health organizations are in the front line of communicating with patient and the value of services provided by health systems for patients is influenced by the nurses behaviors and attitudes [8].

A review of the studies conducted on OCB suggests this concept can be considered as one of the facilitating factors for promotion of care quality in the health and treatment services system. Determining the predicting factors of the OCB and establishing and reinforcing them will result in further reinforcement of the OCB in a hospital and treatment system. Therefore, after reinforcing OCB it is expected that a positive organizational atmosphere, increase in quantity and quality levels of caring, satisfaction of both patients and the personnel, a boost in team work and cooperative decision making, and finally an increase in efficiency and efficacy in the health system will occur [4]. However, the results of studies concerning related and predicted factors regarding OCB are very different. For example, in Chu et al. study [4] job satisfaction (JS) has the role of mediator between OCB and other independent variables, while organizational commitment (OC) did not have this role. Salehi and Gholtash’s study [9] showed that there is a positive correlation between boss JS and OC with OCB. Guangling’s [10] result also showed a sense of justice in the organization through the effect on organizational identity due to strengthening OCB in staff. In contrast to these studies, the research of Alotaibi [11] introduced some controversy about OCB predictors, he found that OCB could not be clearly explained by JC.

As mentioned above, studies have proposed various effective factors, among them the JS, OC and procedural justice (PJ) are referred to here [7, 12]. “JS is a pleasurable or positive emotional state resulting from an appraisal of one’s job or job experiences” [13] and is one of the main components of the organizational health assessment. Its role as an influential factor in strengthening and predicting OCB has been stated in some researches [7, 12]. Moreover, OC is one of the major attitudinal variables in the scope of employment and job. There are several explanations for it, but all the definitions emphasized the individuals’ attachment to their organization [14]. Employee commitment to the organization can be associated with the occurrence and type of OCB [15]. Procedural justice is a type of organizational justice, and in different studies the focus has been on the relationship with OCB. It is defined as the perceived justice about the process that is used to distribute rewards [16].

Literature review about the impact of perceived unfair penalties on OCBs indicated that when supervisors and staff have high perceived organizational justice, they have a greater tendency to engage in OCBs. Dealing fairly with employees can lead to higher commitment and OCBs [15, 16].

Padsakoff in his article reviewed papers on OCB prior to the year 2000. Regarding the serious contradictions in the results of the studies, he contends that these contradictions can be due to influence of cultural context of where the studies had been carried out, thus he makes suggestions on conducting research on factors predicting these behaviors in different countries [17]. From another perspective, considering the aforementioned reviews and based on the influence of OCB in promotion of nursing system, the necessity of taking this concept and its effective factors on emergence and reinforcement into account, it is more commonly observed in the care systems [7].

## The present study

### Aim

This study was conducted to determine the role of JS, OC and PJ in explaining OCB. This study can provide support to create a guideline to help hospital healthcare and nursing managers to further understand how to encourage citizenship behavior among nurses.

### Hypothesis

- Organizational citizenship behaviors have a correlation with JS, OC, and PJ among nurses.
- JS, OC and PJ can explain organizational citizenship behaviors among nurses.

## Methods

### Design and procedures/participants

The current research is a multi-center descriptive-correlational study in which the nurses working in 15 governmental hospitals of Tehran in 2012 were evaluated through a Multi-stage cluster sampling method. Each hospital was considered as one cluster. These 15 hospitals were selected from 25 available hospitals related to Tehran and Iran Universities according to geographic location. Then, based on the total calculated sample volume (400 participants) and the number of nurses in each hospital, the volume proportional to the total number of nurses of that hospital was determined via simple random sampling. Inclusion criteria for the study consisted of holding a bachelor or master’s degree in nursing, a minimum one year job experience, signing an informed consent for participation in the study, and not being in charge of an organizational managerial position, i.e. head nurse, supervisor, or matron, during the questionnaire distribution. Total numbers of nurses in all 25 hospitals related to Tehran and Iran Medical Sciences universities were 4425. The overall sample size was estimated to be 353 participants using Cochran’s sample size formula, then to ensure the cluster coefficient truncation 400 questionnaires were distributed from which 381 were collected. Some incomplete questionnaires were discarded, the remaining 373 were analyzed. Further, to ensure the generalizability power the wards in various shifts (morning, noon, and afternoon) were included in the study.

### Instruments

For the data collection, the following instruments were applied:

1. Demographic questionnaire: including gender and job experience.
2. Padsakoff et al. [18] OCB questionnaire: this questionnaire is composed of 24 questions with 5 degree Likert scale that measures five dimensions of OCB; altruism (five items; α = 0.85), conscientiousness (five items; α = 0.82), sportsmanship (five items; α = 0.85), civic virtue (four items; α = 0.70) and courtesy (five items, α = 0.85). The average inter-correlation between the OCB dimensions was 0.52 [18, 19].This questionnaire was translated to Persian using backward translation method and is used frequently in Iran. Its validity and reliability are confirmed by Gholipour [20] (α = 0.78), Khaef Elahi et al. [21] (α = 0.75), Fani et al. [22] (α = 0.86). Because the Iranian version of the questionnaire was designed for teachers, in the current study, the questionnaire underwent slight changes to suit the nursing society including transforming the terms “teacher” to nurse and “school” to hospital, then the face and qualitative content validity was confirmed by an expert group. Its reliability was calculated through computing internal consistency Cronbach’s alpha coefficient (α = 0.85).
3. OC questionnaire: this questionnaire evaluates different aspects of OC in 7 questions and via 5 degree Likert scale (from completely agree to completely disagree). This questionnaire was applied in a study by Linz [23]. In the current study it was translated into Persian using forward translation and reconciliation of forward translations, then the face and qualitative content validity was confirmed by an expert group. Its reliability was calculated by performing an internal consistency Cronbach’s alpha coefficient (α = 0.85).
4. Alterman et al. JS questionnaire [24]: this tool measures JS with 6 questions and via 5 degree Likert scale (from completely agree to completely disagree). The reliability of this questionnaire was equal to Cronbach’s alpha coefficient 0.79 in Hulpiae et al’s [25] study. Also, the validity of this tool was accepted by Hulpiae et al. [25] by use of exploratory and confirmatory factor analysis. This q0uestionnaire was translated into Persian using forward translation and reconciliation of forward translations, then the face and qualitative content validity was confirmed by an expert group and reliability was calculated by the internal consistency Cronbach’s alpha coefficient (α = 0.77).
5. PJ questionnaire used in this study was extracted from the section of PJ of organizational justice questionnaire developed based on Leventhal et al., conceptualization by Neihoff and Moorman and it has 6 questions and via 5 degree Likert scale (from completely agree to completely disagree). The validity of the mentioned questionnaire was confirmed by others [26–30]. In the current study this questionnaire was translated into Persian using forward translation and reconciliation of forward translations, then the face and qualitative content validity was confirmed by an expert group and reliability was calculated by the internal consistency Cronbach’s alpha coefficient (α = 0.88).

### Data analysis

The data obtained from 373 questionnaires were analyzed by descriptive statistics, Pearson correlation coefficient, comparative mean tests (independent—samples T test, ANOVA) and multiple regression analysis in the SPSS software version 11.

In order to determine the role of independent variables and specify the best predictors in explaining the OCB, multiple regression was applied via Stepwise method based on the *p* value of F (probability of F). For this study, defaults are PIN (0.05) and POUT (0.10). The required defaults for the data to be entered in regression which including: normality of data distribution, Co-linearity statistics, and Durbin-Watson statistic were tested and the results indicated a normal state of data distribution, non-correlation of errors (DW = 1.71) and acceptable coefficients of Tolerance (more than 0.6 for all predictors), and Variance Inflation Factor (VIF) for OC, JS and PJ was 1.64, 1.43 and 1.43, respectively. When statistics of VIF are less than 5 for independent variables, it means there was no co-linearity among them. Briefly, all necessary assumptions for using regression were passed. By taking into account the 5 degree state of questions, based on the calculated mean, the grade 1–1.9 as “undesired”, 2–2.9 as “quite undesired”, 3–3.9 as “quite desired” and 4–5 as “desired” were considered for better interpretation of the data.

### Ethical considerations

Research was continued with the Helsinki Declaration. Research was continued after obtaining approval from the Ethics Committee of Islamic Azad University, Tehran Medical Branch and Tehran University of Medical Sciences research deputy.

## Result

As it was mentioned, the response rate to questionnaires was 93.25 %. About 88.5 % of participants were female, from whom 47.2 % enjoyed job experience of 1 to 5 years. As for their employment situation, 46.9 % were working on contract. The mean, standard deviation (SD) and correlational results of the demographic variables are illustrated in Table 1. The results of t-test showed that the mean score of PJ in the male nurses is higher than female nurses (P < 0.001). In other cases (OCB, JS and OC) there was no significant difference in terms of gender (P > 0.05). Also, the results showed that there were no significant differences among OCB, PJ and OC scores (P > 0.05) in terms of years of experience while JS (P = 0.03) score was statistically significant, at least between the two groups. The Tukey’s Post Hoc test showed that JS score among nurses with job experience between 10 and 15 years was significantly higher than those who have 15 years or more experience. The mean and standard deviation of the main variables under study are illustrated in Table 2. As it is observed, the general mean and the five aspects of OCB achieved higher than average “3” which was evaluated in a “quite desired” state. Among these, the next grade was “Conscientiousness”, which was higher than the other aspects and in the “desired” state and the other four aspects were in “quite desired” state. The mean for perceived PJ from the nurses’ was in “quite undesired” state. The general mean for JS was “quite desired” and most of the participants (57.1 %) had “quite desired” mean for JS. Also, the mean of general grade for OC and JS was achieved in “quite desired” state and maximum individuals (55.6 %) were in a quite desired state in terms of OC. In the next step, the correlation of studied variables was measured via OCB aspects and the results are reported in the Table 2.

**Table 1:** Mean, standard deviations and correlations between demographic variables and the job satisfaction, organizational commitment, procedural justice and OCB dimension among Iranian nurses

	OCB		Job satisfaction		Organizational commitment		Procedural justice	
	Mean ± SD	Sig.	Mean ± SD	Sig.	Mean ± SD	Sig.	Mean ± SD	Sig.
Gender^a^								
Male	3.71 ± 0.36	0.06	3.35 ± 0.84	0.97	3.36 ± 0.71	0.88	3.31 ± 0.89	0.001**
Female	3.83 ± 0.4		3.36 ± 0.8		3.38 ± 0.74		2.79 ± 0.95	
Job experiences^b^								
<5	3.81 ± 0.41	0.94	3.33 ± 0.8	0.02*	3.3 ± 0.75	0.7	2.75 ± 0.93	0.11
5–10	3.83 ± 0.37		3.34 ± 0.77		3.37 ± 0.74		2.88 ± 0.93	
11–15	3.82 ± 0.43		3.64 ± 0.93		3.61 ± 0.76		3.11 ± 1.07	
>15	3.71 ± 0.4		3.36 ± 0.8		3.39 ± 0.8		2.85 ± 0.89	

N = 373

*SD* standard deviation

* Correlation is significant at the 0.05 level

** Correlation is significant at the 0.01 level

^a^Independent samples T test

^b^One Way ANOVA

**Table 2:** Mean, standard deviations and Correlations for the job satisfaction, Organizational commitment, procedural Justice with OCB dimension among Iranian Nurses

Item	Mean	SD	1	2	3	4	5	6	7	8	9
1. Job satisfaction	3.36	0.8									
2. Organizational commitment	3.37	0.76	0.52**	–							
3. Procedural justice	2.85	0.96	0.42**	0.53**	–						
4. OCB overall	3.81	0.4	0.27**	0.42**	0.12*	–					
5. Altruism	3.91	0.6	0.28**	0.26**	0.14*	0.64*	–				
6. Conscientiousness	4.21	0.71	0.006	0.10*	0.22**	0.59**	0.18**	–			
7. Sportsmanship	3.88	0.9	0.023	0.065	0.12*	0.53**	0.05	0.59**	–		
8. Civic virtue	3.37	0.72	0.21**	0.36**	0.29**	0.44**	0.31**	0.07	0.12*	–	
9. Courtesy	3.75	0.52	0.29**	0.41**	0.22**	0.75**	0.41**	0.19**	0.12*	0.29**	–

N = 373

*OCB* organizational citizenship behaviors

** *p* < 0.01, * *p* < 0.05

* Correlation is significant at the 0.05 level (2-tailed)

** Correlation is significant at the 0.01 level (2-tailed)

As shown in Table 3, the results obtained from regression indicated that OC and PJ have been entered to the model and JS and demographic variables were omitted. Organizational commitment (β: 0.421) has the most significant impact on OCB. In the first step, OC was entered to the model as the strongest predictor in OCB. It is shown that 17 percent of the variation in OCB could be explained by OC. In the second step, PJ was added to the model (standard beta: 0.136), beta value for OC changed to 0.493 and R square was increased to 19 % in regression equation. This means that OC and PJ have significant impact and contribution in explaining OCB while other variables remained constant.

**Table 3:** Coefficient of multiple regressions and ANOVA results

Step/predictor(s)	β	t	sig.	R square	F	sig.
1						
(Constant)		35.55	<0.001	0.17	79.956	<0.001
Org. commitment	0.421	8.942	<0.001			
2						
(Constant)		35.72	<0.001			
Org. commitment	0.493	8.96	<0.001	0.19	43.314	<0.001
Procedural justice	0.136	2.48	0.014			

Dependent variable: OCB

Method: stepwise (criteria: p-value to enter ≤0.05, p-value to-remove ≥0.1)

JS have not been entered to the equation, which means this variable did not have default criteria to inter to the model and its impact on exhibiting OCB was not significant.

## Discussion

The mean of grade for OCB in this study was achieved in a quite desired state and as it was mentioned, the maximum grade was achieved for Conscientiousness. Similar to our finding, the studies conducted by Jafari et al. [31] and Yazdani et al. [32] regarding Iranian nurses indicate comparable results, the difference being that maximum points in Jafari et al. were obtained for Conscientiousness and in Yazdani et al. they were obtained for Altruism [31, 32]. In Altuntas & Baykal’s study [33] performed in Istanbul, Turkey (2010) on 482 nurses working at 11 medical centers, the mean OCB scores other than Sportsmanship have a higher than average level and the maximum grade was calculated for Conscientiousness. Blau et al’s study [34] regarding Sportsmanship behaviors in hospital nurses in Oman indicated that the mean of grades for nurses in respected questionnaires in both job and organizational factors was higher than the overall mean.

Based on the literature, many contextual and environmental factors influence the employees’ OCB. Among these factors, diversity of different hospital wards and various dominant conditions, different job difficulties at wards and hospitals, differences in the job description for nurses in different countries, differences in the rate of wages and variables related to the society and culture of each country are critical factors and have caused differences in the rate of nurses’ OCB [17, 18]. These differences have motivated new questions, ambiguities and variables regarding OCB based on Padsakoff’s views, while providing an opportunity to compare these variables to differing degrees [17, 18]. High level of OCB among nurses in a hospital would transform the work place to an attractive and active environment and as a result the hospital could maintain a better performance in providing care and health services by employing more efficient work forces [35].

This study result showed that male nurses compared to female, have a better understanding of PJ from their head nurses and other nursing managers. But in other cases (OCB, JS and OC) there was no significant difference in terms of gender. In contrast to the present study, the Royal study [36] about Virginia nurses showed that there was no relationship between gender and JS and organization commitment scores. In the field of OCB and attitude of staff, gender is one of the variables that is related to many other variables, it is also mentioned as the most important demographic and moderator variable. But the role of gender differences in OCB, JS and OC remains a challenging issue [37].

The results also showed that there were no significant differences about OCB, PJ and OC scores in terms of years of experience while JS scores among nurses with job experience between 10 and 15 years were significantly higher than those who have 15 years or more experience. Bidarian et al’s study [38] determined that increases in the years of staff experiences improve the OCB and perceived organizational justice. The Royal study [36] also showed that years of experience had significant correlation with JS and emotional organization commitment.

The obtained results indicate that OC alone explains 17 % of OCB which is statistically significant, therefore, it can be said that among the studied variables in this research, OC was the strongest predictor of nurses’ OCB and this rate increased to 19 % by adding perceived procedural justice. In other words, these two variables together can explain 19 % OCB, and other variables including demographic variables and JS were omitted from the model. Contrary to the results of other studies, JS in predicting nurses’ OCB was not statistically significant. In other words, JS did not have an independent and significant portion in explaining nurses’ OCB in the current study. The result of a study by Salehi and Gholtash [9] indicated that JS, OC, and job burnout explain 2 % of OCB variation. In present study, OC was the only variable that had direct and positive effect on OCB, also, JS did not have direct effects on OCB [9]. In the study by Chu et al. [4], JS, perceived procedural justice, supervisor support and job involvement were the variables predicting Taiwani nurses’ OCB and, unlike the current study, OC could not significantly explain OC [4]. Using different methods of research in terms of data collection, applying different questionnaires, and contextual differences and different job values in Taiwan compared to those in Iran explains the reasons behind the differences between the two studies. The results of the study by Guangling [10] in a Chinese company indicated that PJ alone can predict 17 % of employees’ OCB. He explains the mechanism of the relation between these two variables as: when an employee feels that the things he achieves from the organization are more than the work he does, performing the tasks out of duty would be considered as compensation. In return, when the person feels that the job he does is more than what he is receiving from the organization, he will set aside part of the responsibility of his duty or even leave the organization [10].

Tabarsa et al’s study [35] regarding the effect of OC variables, JS, organizational trust, distributive justice, interactional justice, and PJ on OCB of nurses in Iran indicated that among the above mentioned variables only PJ and OC can directly explain the OCB. In their study, JS did not directly affect the OCB, but it influenced OCB through OC. These results, to some extent, confirm the results of the current study [35]. Strugar [39] in his study showed that OC and PJ have a significant role in explaining the OCB, and other variables lacked significant predicting power. However, all of the four variables JS, OC, PJ and distributive justice had significant correlation with the OCB [39]. Freshwater’s study [40] in an industrial institute indicated that when the role of other variables is controlled, JS and organizational justice cannot significantly explain the OCB and R square change is not statistically significant. However, the results of studies conducted regarding the factors predicting the OCB are diverse and sometimes contradictory.

In the current study, despite existence of significant correlation between JS and the OCB, this factor did not specifically explain the significant variation in multiple regression. In Alotaibi ’s [11] study in Kuwait, Stugar’s [39] in the USA and Freshwater’s [40] in line with the current study, JS could not significantly predict the OCB [11, 39, 40]. Alotibi [11] believes that meaningful explanation of OCB through a series of studies may result from overlap and correlation of JS with other factors such as organizational justice, the role of which is not controlled in some of these researches. In this study there was no co-linearity between three independent variables, so the obtained results are not affected by co-linearity and it can be said that the results are reliable. In contrast to this study, Carver et al. [41] and Gutierrez et al. [42] indicated that global JS is a predictor for OC in nursing faculties in academic organizations [41, 42].

Based on the results of the current study, the necessary grounds for creating commitment among the nurses in a hospital are created through their participation in decision making, considering various welfare and educational needs of competent nurses, individual and characteristic differences, justifying the nursing personnel regarding their job decisions, head nurses and managers understanding their family and job conditions [43] and also providing in-time support for nurses in challenging conditions, as meeting the occupational and non-occupational needs of employees are mentioned as factors contributing to an increase in their commitment to the organization [44]. On the other hand, equal distribution of facilities, justice in regulating working shift plan, equal procedure for annual promotion, clear criteria for organizational promotion and also proportion of the receiving salary and bonus with the person’s efficiency are suggested to be considered in order to have a positive perception about PJ so that by creating emotional and normative commitment in nurses toward the hospital they work for and positive understanding of procedural justice, the grounds for emergence of citizenship behaviors, using maximum potential of personnel and as a result improvement and promotion of care condition can be provided, while preventing resignation and leaving service by the staff.

## Conclusion

OC was the most powerful predictor of nurses’ OCB and perceived PJ was the second variable predicting this type of organizational behavior in the current study. The grounds for emergence of OCB require strong roots and back-ups to cause emergence of such behavior by the hospital employees and nurses are no exception. Provided that these grounds be recognized and improved, these behaviors will be accepted as a component of the belief system among the employees in an organization and through which the prosperity will probably occur. This study highlights and illustrates the role of OC and perceived PJ in explaining organizational citizenship behavior among Iranian nurses and does not prove JS as an independent predictor of nurse’s OCB, thereby offering adjusted intervention perspectives in health care system.

OCB is a variable that is dependent on context and has multi-aspects and other factors such as participant characters, culture, trust in supervisor, and organization can influence that. By taking into account the methodology of current study, controlling all these factors in this study is not possible and thus performing prospective studies, qualitative research methods and deep interviews which can better explain this variable are proposed.

## Abbreviations

OCB: organizational citizenship behavior
SPSS: Statistical Package for the Social Sciences
OC: organizational commitment
JS: job satisfaction
PJ: procedural justice
